# Primary Hydatid Cyst of the Axillary Region: A Case Report

**DOI:** 10.1155/2012/362610

**Published:** 2012-12-17

**Authors:** Mehrangiz Zangeneh, Mahmood Amerion, S. Davar Siadat, Mohsen Alijani

**Affiliations:** ^1^Department of Infectious Diseases, Islamic Azad University, Tehran Medical Branch, Tehran, Iran; ^2^Department of General Surgery, Islamic Azad University, Tehran Medical Branch, Tehran, Iran; ^3^Department of Microbiology, Pasteur Institute, Tehran, Iran

## Abstract

*Introduction*. Hydatid disease is a disease caused by the cestode *Echinococcus*. *Echinococcus granulosus* is the most common *Echinococcus* species affecting human. It may affect any organ and tissue in the body, most in the liver and lung. Disease is endemic in some regions of the world, and is common in Iran. Primary hydatid cyst of the axillary region is an unusual and rare localization of hydatid disease. So far, only sixteen cases have been published in the all medical literature. *Case Report*. Herein, we present a 33-year-old woman because of a mass in the axillary region of four months duration. Axillary ultrasonography showed a thick wall cystic lesion. No abnormality was found in mammographic examination of either breast, or in abdominal ultrasonography and chest X-ray. The mass was excised for pathological examination that showed a typical laminated membrane of hydatid cyst. Postoperative IgG- ELISA serology in this case was negative. Based on pathology an axillary hydatid cyst was diagnosed. *Conclusion*. Hydatid cyst should be considered in endemic areas in patients presenting with a soft tissue mass in the axillary region.

## 1. Introduction

In human, three forms of echinococcosis are known to occur: cystic echinococcosis, caused by *Echinococcus granulosus*, alveolar echinococcosis, caused by *Echinococcus multilocularis*, and polycystic Echinococcosis due to *Echinococcus vogeli* or *Echinococcus oligarthrus*. Hydatid disease is a parasitic disease usually caused by the larval stage of a small zoonotic tapeworm primarily found in dogs *Echinococcus granulosus* [1]. *Echinococcus granulosus* (cystic echinococcosis) is the most common species, *E*. *granulosus* has worldwide distribution and is endemic in many countries, especially the Mediterranean region, Australia, South America, the Middle East, South Africa, and Eastern Europe [1–3]. Iran is also an endemic area for hydatid diseases, the prevalence of cystic echinococcosis in humans detected by ultrasound ranges from less than 0.5% to 1.5%. Human seropositivity was greater than 5% in the west and southwest of the Islamic Republic of Iran, with 2–18% seroprevalence in nomadic groups [4, 5]. The hydatid cysts of *E*. *granulosus *tend to form in the liver (50% to 70% of patients) or lung (20% to 30%) but may through the capillary systems reaches the general circulation and passes to all viscera and soft tissues. For this reason, hydatid cysts may arise in atypical sites such as the brain, heart, orbit, urinary bladder, chest wall, subcutaneous tissue, tibia, parotid gland, breast, cervicofacial region, thyroid, and in any organ of the body (10%) [1, 3, 6, 7]. The diagnostic methods include imaging techniques, mainly X-ray for lung echinococcosis, ultrasound and computed tomography examination for abdominal echinococcosis and other affected organs, and immunodiagnostic tests (enzyme-linked immunosorbent assay (ELISA), IFAT, and immunoblot) for confirmation. IgG-ELISA is about 90% sensitive for liver cyst infection but less sensitive for lung (80%) or other organ involvementand (90%), and its specificity is 90% [1].

Symptoms are often absent, and in many cases infection is detected only incidentally by imaging studies. Its symptoms depending on the host organ, location, its effect on adjacent structures, complications due to rupture, secondary infections, and immunological reactions caused by the cyst [1, 5]. Primary axillary hydatid disease is rare even in the endemic regions, we could find only sixteen case reports in the literature [3, 6, 8–21]. In this paper, we report a case of a primary hydatid disease which originated in the subcutaneous tissue in the right axillary region in a 36-year-old woman.

## 2. Case Report

**Figure 1:**
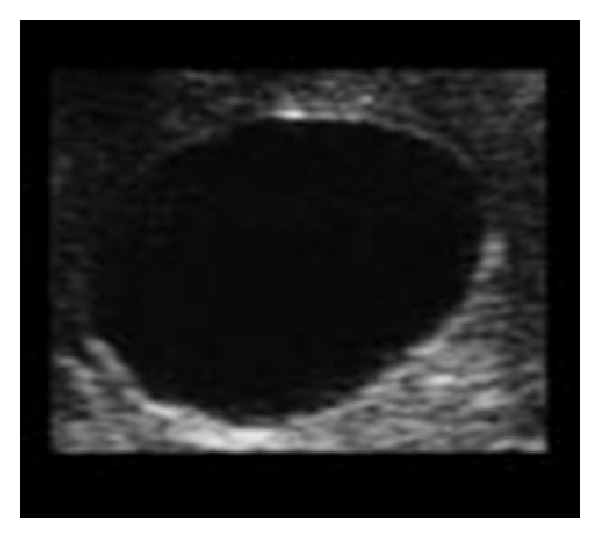
Ultrasound image of axillary cyst.

**Figure 2:**
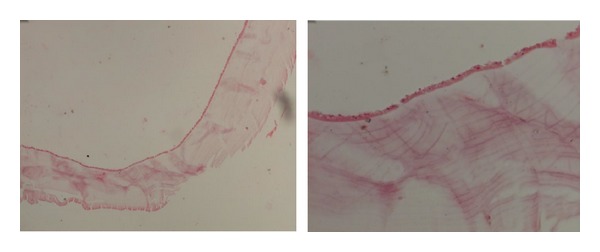
Section of cyst wall showing germinal epithelium and underlying laminated membrane (*haematoxylin-eosin stain,* magnification ×10).

A 33-year-old woman in 2010 was presented to our hospital with painless right axillar swelling of four-month duration. She had no history of breast mass, fever, other symptoms, or other mass, and also had no history of hydatid cyst. On physical examination, a firm to hard 5 × 5 cm mobile mass was noted in the right axilla. Laboratory investigations were done which revealed normal blood counts and biochemical result. Ultrasound showed a 5 × 5 cm thick wall cystic lesion comparable with type CE1 of WHO classification (Figure 1). No abnormality was found in mammographic examination of either breast. In this case it was difficult to differentiate a cyst with uncharacteristic imaging findings (type CE1 of WHO Classification based on ultrasound) from simple subcutaneous cyst, haematoma, necrotic tumor, or lymph node based on ultrasound. Under general anesthesia, the entire cyst was excised without rupture and sent for histopathological examination. On macroscopic examination, a unilocular cyst with a hard fibrotic wall was seen. Microscopic examination revealed the presence of a typical laminated membrane of hydatid cyst (Figure 2). The hydatid IgG-ELISA test in our case was negative. Radiographs of chest, spine, and long bones were done for evaluation of hydatid in organs and all were normal. Abdominal ultrasound examination was normal. The patient was discharged from the hospital with albendazole 400 mg twice a day for four weeks. During one-year followup, examinations since her surgery have shown no relapse of hydatidosis.

## 3. Discussion

Hydatid disease is a common clinical pathology in many parts of the world. The main species pathogenic for humans in Mediterranean and Southern European countries is *Echinococcus granulosus* [1, 2]. Hydatid cysts most often develop in the liver and lung. primary axillary hydatid disease is rare, as shown in Table 1, we could find only sixteen previous case reports in the medical literature [3, 6, 8–21] (Table 1).

Hydatid cysts grow 5 to 10 cm in size within the first year and can survive for years or even decades. The cyst may be present for many years in the organ in which it is located with no clinical symptoms or signs, and sometimes it may exhibit clinical symptoms depending on the size and location of the cyst and the pressure of the growing cyst [1, 6]. The sonographic and tomographic appearances of subcutaneous hydatid disease such as axillary hydatid cyst are similar to those in other organs. Hydatid cyst may be unilocular at earlier stages, whereas older cysts are usually multilocular. They may either be made up of daughter cysts or have a solid appearance made up of multiple septated cysts. However, hyperintense hydatid cysts in the axillary region can be misdiagnosed as a soft tissue tumor or lymphadenopathy. As Iran is an endemic area for hydatid disease, this should be in the differential diagnosis for patients presenting with tissue masses including haematoma, abscess, sarcoma, lymphadenopathy, breast cancer, or metastatic lesions [1, 3, 18]. Infection is suspected based on imaging studies (ultrasonography, CT, and MRI), and it may be confirmed by a specific enzyme-linked immunosorbent assay (ELISA) and Western blot serology. Ultrasonography is an easy available, and affordable with high diagnostic sensitivity imaging test for screening of hydatid cyst in endemic areas and in family members based on WHO classification. The sonographic imaging in our case showed a simple cystic lesion comparable with type CE1 of WHO Classification and did not confirm a diagnosis of hydatid cyst; it is difficult to differentiate a cyst with uncharacteristic imaging findings from simple subcutaneous cyst, haematoma, necrotic tumor, or lymph node based on ultrasound. Immunodiagnostic tests (enzyme-linked immunosorbent assay (ELISA), IFAT, and immunoblot) are used for confirmation. IgG-ELISA is about 90% sensitive for liver cyst infection but less sensitive for lung (80%) and other organ involvement (90%), and its specificity is 90% [1]. Postoperative IgG-ELISA test in this case was negative. Abdominal ultrasonography and a plain chest radiography are mandatory to detect liver and lung involvement. Chest X-rays and imaging studies showed no other involvement in our patient. In routine practice, the accurate diagnosis in patient with soft tissue hydatidosis is frequently delayed until histopathologic examination after surgery. In this case, for accurate diagnosis, the entire cyst was excised. 

Today, treatment options for CE (cystic echinococcosis) include surgery, PAIR (puncture, aspiration, injection, and reaspiration), and chemotherapy. Optimal treatment of hydatid cysts is surgical resection to remove the cyst. The main purpose of the surgery is to prevent the patient from complications such as compression of surrounding structures, infection or rupture of the cyst [1, 7]. Total cystectomy with fibrous adventitia which allows removal of all parasitic elements without spillage of the contents of the cyst, is curative treatment for soft tissue hydatidosis. We performed total cystectomy without rupture in spite of the anatomical distortion of the axillary region. Antiparasitic medication is widely used postoperatively and preoperatively for the purpose of cyst size reduction and to limit the risk of intraoperative dissemination of daughter cysts. All patients are treated with albendazole 10 mg/kg/day for at least two weeks preoperatively, and this is continued postoperatively for four weeks [22]. However, the experience with scolicidal agents such as albendazole (400 mg/kg) and praziquantel (50 mg/kg) or a combination of these drugs in the treatment of soft tissue hydatid disease is very limited and results of the medical treatment in the hydatidosis are unclear [23, 24]. We gave the patient albendazole 400 mg twice a day for four weeks postoperatively. 

In conclusion, hydatid disease is a widespread public health problem in developing countries especially in endemic regions; therefore, it should be considered in the differential diagnosis of a palpable mass in the axillary region.

## Figures and Tables

**Table 1:** Reported cases of hydatid cysts of the axilla.

Author	Year	Age/gender	Origin of cysts	Daughter cysts	Organ involvement	Screening	Follow
Thomson [8]	1899	NA	Axillary	NA	NA	NA	NA
Lamotte et al. [9]	1967	32/M	Right axillary vein	Multiple	None	CXR	NA
Zamfir et al. [10]	1997	11/F	Left neurovascular	None	pulmonary	CXR, US	NA
Navarro Martín et al. [11]	1998	84/M	Subcutaneous	Multiple	None	CXR, US	NA
Mayol Martínez et al. [12]	1994	67/F	Muscle	Multiple	None	CXR,US	NA
Ekrem Unal et al. [13]	2001	53/F	Right pectoral M	None	None	MRI, CT	9 months
Dilege et al. [14]	2003	15/F	Right axillary region	NA	Pulmonary	CXR, CT	NA
Losanoff et al. [15]	2004	38/M	Subcutaneous tissue	NA	None	NA	NA
Sain Guven et al. [16]	2004						
Borovik et al. [17]	2006	31/F	Left axillary	Multiple	None	CXR, CT	6 months
Singh et al. [18]	2009	28/M	Left lateral chest wall	NA	None	CXR, CT	8 years
Ünalp et al. [3]	2011	48/F	Left axillary fossa	Multiple	None	CXR, US, CT	6 months
Ozsoy et al. [6]	2011	45/F	The axillary region	None	None	CXR, US, CT	1 year
Ruso et al. [19]	2011	84/F	Cervicoaxillary region	Multiple	None	CXR, US, CT	5 years
Arsalane et al. [20]	2012	43/M	Left axilla	Multiple	None	CXR, US, CT	NA
Saylam et al. [21]	2012	36/F	Right axillar	None	None	CXR, US	17 months

F: female, M: male, CT: computed tomography, CXR: chest X-ray, LP: laparoscopy, RS: radionuclide scintigraphy, US: ultrasonography, NA: data not available, MRI: magnetic resonance Imaging.
